# Feedback Focused: A Learner- and Teacher-Centered Curriculum to Improve the Feedback Exchange in the Obstetrics and Gynecology Clerkship

**DOI:** 10.15766/mep_2374-8265.11127

**Published:** 2021-03-25

**Authors:** Natasha R. Johnson, Andrea Pelletier, Celeste Royce, Ilona Goldfarb, Tara Singh, Treven C. Lau, Deborah D. Bartz

**Affiliations:** 1 Clerkship Director, Department of Obstetrics and Gynecology, Harvard Medical School/Brigham and Women's Hospital; 2 Statistician, Department of Obstetrics and Genecology, Harvard Medical School/Brigham and Women's Hospital; 3 Clerkship Director, Department of Obstetrics and Gynecology, Harvard Medical School/Beth Israel Deaconess Hospital; 4 Associate Clerkship Director, Department of Obstetrics and Gynecology, Harvard Medical School/Massachusetts General Hospital; 5 Clerkship Director, Department of Obstetrics and Gynecology, Harvard Medical School/Cambridge Health Alliance; 6 Clerkship Director, Department of Obstetrics and Gynecology, Harvard Medical School/Massachusetts General Hospital; 7 Associate Clerkship Director, Department of Obstetrics and Gynecology, Harvard Medical School/Brigham and Women's Hospital

**Keywords:** Feedback, Formative Feedback, Learner Training, Faculty Development, Dual Investment

## Abstract

**Introduction:**

Learners consistently report insufficient feedback, despite interventions to improve the quantity and quality of feedback. Effective feedback requires a dynamic partnership and a trusting relationship between students and teachers.

**Methods:**

We developed and implemented a faculty and student program called Feedback Focused on the OB/GYN clerkship with learner- and faculty-centered teaching materials. We evaluated the curriculum's impact on the frequency and quality of feedback exchange through comparison of end-of-clerkship evaluations before and after implementing the Feedback Focused program and assessed student satisfaction from written responses on clerkship evaluations.

**Results:**

A total of 1,912 feedback folio entries were recorded during the curriculum timeframe, representing an average of 19 entries per student. Of students, 85% turned in their feedback folios at the end of the clerkship. There was a marked increase in reported frequency of feedback with the initiative, with 28% of students reporting receiving feedback four or more times per month before the start of our program, compared to 64% after its completion. The percentage of students who reported faculty provided direction and constructive feedback *always* or *very often* remained roughly the same before and after the program (69% vs. 70%, respectively). Over 60% of students provided positive feedback on written open response questions.

**Discussion:**

We successfully developed and implemented a multipronged approach to effectively change the learning environment culture within our OB/GYN clerkship program. Our evaluation demonstrated that the Feedback Focused program was well received and increased frequency of feedback.

## Educational Objectives

By the end of this activity, faculty and student learners will be able to:

1. Perform effective feedback exchanges.
2. Give and receive actionable, constructive feedback.

## Introduction

The process of professional learning within medical school is predicated on the effective transfer of feedback as a means of highlighting the gap between observed performance and desired performance in medical trainees. Constructive feedback, defined as feedback based on direct observation to improve the trainee's performance,^1^ is required for students to improve their skills, meet professional goals, and, ultimately, to deliver better care to patients. While learners have reported a desire for more feedback, they consistently report insufficient feedback,^2–4^ or do not recognize feedback when they received it,^3,4^ despite a recent focus on faculty trainings and other interventions to improve the quantity and quality of feedback. Effective feedback requires a dynamic partnership and a trusting relationship between students and teachers^5,6^ where all parties have an ongoing exchange.^7^

Prior research has taken a unidirectional approach to improving feedback.^8–10^ Several curricular changes and interventions focus on training faculty, assuming the challenge is with the feedback giver. Trainings typically focus on providing nonthreatening feedback that is timely, nonevaluative, based on specific observation, and provides a plan for improvement.^7,11–13^ In general, faculty report providing feedback at much higher rates than learners report receiving it.^1,14^ Addressing this disconnect is a constant challenge for medical faculty. Faculty may be afraid to provide negative feedback^6,7^ or just do not have time to give formative feedback.^15^ Feedback curricula published previously in *MedEdPORTAL* have utilized video vignettes to train faculty to develop a shared mental model of feedback^16^ or incorporated the use of a direct clinical observation tool.^17^

Other published unidirectional feedback curricula have focused efforts on the learner, teaching recognition, solicitation, and utilization of constructive feedback.^18,19^ Many argue that learners take a passive role in the physician-student feedback dyad and require proper training on soliciting, recognizing, and utilizing feedback in medical education.^4–5,9,20^ Additionally, students may not recognize feedback when they receive it or may have low self-esteem or overestimate their abilities,^4^ thus affecting their perceptions of the feedback they do get.^4–5,21^ In addition, learners have reported a preference for feedback that is interactive and self-initiated,^9^ yet many do not understand how to incorporate these techniques into the clinical training environment.^22^ Milan and colleagues showed that with a specific learner training intervention, students had more positive attitudes about obtaining feedback and increased frequency of feedback-seeking behavior.^9^

While unidirectional curricula are invaluable to the medical education community, they do not provide tools to simultaneously train both faculty and students to collaborate in the feedback process. Despite the importance of isolated faculty or learner-centered approaches, neither one alone accomplishes the goal of improving quality and quantity of the trainer-trainee dyad around the successful transfer of information required for high-quality feedback sessions. Moreover, training only one side of this partnership could have a deleterious effect.^20^ Thus, our learner-centered training focused on teaching students techniques to effectively solicit feedback related to their learning goals while simultaneously training faculty.

Unpublished curriculum quality assessment results from Harvard Medical School's (HMS) AAMC graduation questionnaire (GQ) and student evaluations of the OB/GYN clerkship identified, “Faculty providing direction and constructive feedback,” as the area for greatest improvement in the learning environment of the OB/GYN clerkship. To address these results, we created a learner and faculty dual investment, in which both parties in the dyad invested in the feedback program in an initiative we coined Feedback Focused. The objective of our dual investment initiative was to change the culture of our learning environment to improve both the quantity and quality of feedback provided to our principal clinical experience (PCE) students. We hypothesized that the transfer of constructive feedback would improve as measured by the students’ perception of the learning environment on postclerkship evaluations. Within this publication we describe the design, implementation, and evaluation of the Feedback Focused initiative during the OB/GYN clerkship.

## Methods

### Setting

HMS has four main affiliated hospitals which train second-year students during their OB/GYN core clerkship as part of the PCE: Beth Israel Deaconess Medical Center (BIDMC), Brigham and Women's Hospital (BWH), Massachusetts General Hospital (MGH), and Cambridge Health Alliance (CHA). This OB/GYN clinical training is overseen by the HMS OB/GYN clerkship committee which meets quarterly and ensures similar learning environments and clinical experiences to all HMS students, regardless of training site. This Feedback Focused intervention was developed by this HMS OB/GYN clerkship committee and initiated at all four PCE training sites simultaneously. This project was approved by the HMS educational scholarship for the program in medical education as quality improvement on September 11, 2018. It was also approved by the Partners Internal Review Board on November 21, 2018 (protocol #2018P002335).

### Development and Implementation

#### Faculty-centered training

We first conducted a literature review regarding best practices in the provision of constructive feedback to medical trainees^7,10,18–19,21,23–25^ in order to inform the development of the program content including: the faculty development content and roll-out (Appendices A and B); lanyard badges for faculty to wear at all times (Appendix C); feedback outcomes to be captured and monitored (Appendix D); slogan and logo (Appendix E); and poster advertisements for all clinical spaces (Appendix F). The “OB/GYN PCE is Feedback Focused” branding of the program that was meant to affect the overall culture of our respective clerkships.

To introduce faculty to the Feedback Focused program, clerkship directors at each site spoke at grand rounds, faculty meetings, departmental division meetings, resident didactics, educational retreats, and one-on-one meetings with individual faculty to make sure everyone within the departments was included. These faculty development sessions, detailed in Appendix A, described the scope of the problem and impetus for creating the feedback program, including the importance of a positive learning environment. To provide motivation for improvement, we also shared the low ratings on the statement, “Faculty provide direction and constructive feedback,” reported by our students on the AAMC GQ and HMS OB/GYN clerkship end-of-clerkship evaluations. We outlined the initiative's goals and requirements of the program including students’ roles in soliciting and documenting feedback and the clerkship tracking mechanism. The main focus of the training was to encourage faculty to provide actionable constructive feedback based on direct observation, a type of feedback that has been shown to improve performance, as opposed to compliment-style feedback, which students do not find helpful.^1,20^ Teaching faculty to label the feedback was emphasized, given the disconnect in the literature between faculty-reported rates of providing feedback compared to learner-reported rates of receiving it,^1,14^ and the fact that learners do not always recognize when they are receiving feedback.^3,4^

The faculty teaching points were reinforced through a 7-minute Association of Professors and Gynecology Organization video “Effective Preceptor Series*:* Providing Educational Feedback” (Appendix B) shared via email and viewed independently. The viewing of this video was not mandated but was strongly encouraged by the clerkship directors. In addition, the week prior to the start of the initiative, the clerkship coordinator provided faculty with laminated teacher tips cards (Appendix C) to wear on their identification badge lanyards. One side of the card provided best practices for giving actionable, constructive feedback. The other side included helpful verbal scripts for initiating effective feedback. Lastly, we created and printed posters to place in clinical teaching settings (including ambulatory clinics, labor and delivery, and student work room) to remind faculty and students of the Feedback Focused program (Appendix F). Email reminders were sent by the clerkship coordinator to all faculty, fellows, and residents at all sites every 6 weeks at the start of each clerkship throughout the year-long program to emphasize the program's importance in effecting culture change.

#### Learner-centered training

As with the faculty, HMS student training, detailed in Appendix G, consisted of providing background information and data for the scope of the problem and impetus for the program; however, for the learners we stressed that our motivation for improvement was based on student feedback that, “Faculty provide direction and constructive feedback” on the HMS OB/GYN end-of-clerkship evaluation.

Clerkship directors introduced the Feedback Focused program at the beginning of each clerkship rotation during student orientation at all four PCE sites. Using the PowerPoint (Appendix H) we introduced our HMS “OB/GYN PCE is Feedback Focused” logo and brand and presented the remainder of the slides which focused on eliciting, interpreting, and utilizing feedback during the clerkship program and beyond. Laminated learner tip cards (Appendix I) and feedback folios (Appendix D) were distributed at clerkship orientation. The learner tip cards described evidence-based best practices for eliciting and receiving feedback.^18^ One side of the card emphasized the importance of asking (ASK) for feedback early and often. The other side of the tip card had the acronym READY: reflect on performance, engage in the process of feedback, aspire about skills to develop, define areas for improvement, and you - responsibility for growth is yours.

To encourage the incorporation of daily feedback into learning, students were reminded weekly by clerkship directors to solicit and record feedback at a minimum of once daily. The feedback folio (Appendix D) was developed as a pocket handbook in which students were asked to record the setting, nature, and source of all feedback. In addition, students recorded if the feedback was scheduled, timely, and perceived as constructive, their satisfaction with the feedback, and what behavior they would change, if any, based on the feedback. These variables were included on the feedback folio to provide insight into the feedback characteristics that might be correlated with higher levels of satisfaction with feedback and with intended behavior change.^25^ Students reviewed the feedback folios individually with their clerkship director at mid- and end-of-clerkship feedback sessions. The feedback folios were collected at the end-of-clerkship feedback session and students were asked to rate their overall satisfaction with the feedback initiative on end-of-clerkship evaluations (Appendix J).

### Analysis and Program Evaluation

#### Student end-of-clerkship evaluation

To determine if this initiative translated into an improvement in the HMS OB/GYN PCE evaluation deficiency, we obtained deidentified, aggregate, end-of-clerkship evaluation data from HMS for the academic year prior to the start of the Feedback Focused initiative. CHA PCE folios were collected although not included in our data analysis as they have a longitudinal integrated curriculum structure which was different from and incomparable to the traditional block clerkships at the other three hospital sites.

We compared student responses from pre- and postintervention for the following questions: “How often did faculty provide direction and constructive feedback?,” “From whom did you receive feedback?,” and “How many times per month did you receive feedback?” In addition, we added questions to the end-of-clerkship evaluation to assess student satisfaction with the frequency, quality, and incorporation of constructive feedback into the PCE rotation (Appendix J).

#### Student folios

At the conclusion of the OB/GYN PCE, de-identified data from the feedback folios were entered into REDCap and exported into Stata (version 15.0), and descriptive analyses were performed. We coded open-ended responses to the question, “What do you think about the overall feedback program on this clerkship?” on the end-of-clerkship evaluations using content analysis to determine the presence or absence of certain themes.

#### Faculty focus groups

We conducted a focus group after the Feedback Focused program ended to acquire faculty perspectives on changes in quantity and quality of feedback as well as feedback culture change in the department due to the curriculum initiative. We sent email invitations to 10 faculty members from the three sites. Faculty were targeted based on their involvement in leading medical student didactics, being recognized for teaching, or participating in core preceptor programs. The study statistician moderated the focus group which was held via Zoom and lasted 40 minutes. The meeting was recorded. Two independent reviewers (Andrea Pelletier, Natasha R. Johnson) watched the recording, took notes, summarized the discussion surrounding each question, and identified themes using content analysis. Focus group questions were included in Appendix K.

## Results

### Participation

All HMS students entering the OB/GYN PCE at BWH, MGH, BIDMC, and CHA beginning July 2, 2018 and ending May 30, 2019 were included in this curriculum (a total of seven 6-week rotations for BWH, MGH, and BIDMC; CHA has a yearlong longitudinal integrated curriculum^26^). All faculty in the OB/GYN departments at the four sites were included in the faculty initiative. In total, there were 156 HMS students and 205 faculty.

After excluding the CHA participants, a total of 144 students participated in the PCE during the feedback program period. We collected student feedback folios from 122 (85%) students and evaluations from 138 (96%) students, evenly distributed amongst the three hospital sites where data were collected (BWH = 46, MGH = 47, BIDMC = 45).

### Student End-of-Clerkship Evaluation

From end-of-clerkship student evaluations collected after implementation of the Feedback Focused program, 88% of students reported the feedback they received to be constructive *always* or *often*, 84% reported feedback amount was *adequate* (compared with *not enough*, 13%; or *too much*, 3%; and 64% said they received feedback four or more times per month). When asked how often faculty provided direction or constructive feedback, 70% of students responded *always* or *very often*.

As compared to end-of-clerkship student evaluations from the academic year prior to the implementation of the Feedback Focused program, there was a marked increase in reported frequency of feedback at all sites. Overall, the percentage of students who reported receiving feedback four or more times per month increased from 28% before the initiative to 64% after the initiative. The percentage of students that reported faculty provided direction and constructive feedback *always* or *very often* remained roughly the same before and after implementing the program (69% vs. 70%, respectively).

### Student Folios

A total of 1,912 feedback entries in the feedback folios were recorded, representing an average of 16 entries per student (*SD* = 6) over approximately 30 clinical days. Approximately half of students responded to the open-ended question asking for general feedback about the program. Themes identified from the content of the open-ended question included: “positive overall,” “neutral overall,” “negative overall,” “feedback folio feedback,” “lack of connection with faculty,” and “criticism of residency teaching.” Over 60% of students who responded had positive feedback. Examples of these comments were:

• “I liked how we had to proactively seek feedback because in the future that will be the case. I also liked how we received both in-the-moment and written feedback.” (Student 62)
• “I liked the feedback folio. It helped remind me to ask for feedback and also since I knew that the whole department was very feedback-focused it made asking for feedback less awkward.” (Student 77)

Criticisms of the program included lack of connection with faculty, not enough feedback from residents, not enough constructive feedback, and feedback too artificial or forced. Some examples included:

• “I think it is great that this is important to the clerkship, but you can't give feedback when you don't get to build meaningful relationships with faculty or house staff.” (Student 20)
• “The idea is good, but we really don't get much feedback from residents or attendings (probably because we do not spend enough time with any one person) and receiving a ‘good job’ doesn't help us be better or work on anything specific.” (Student 82)

In addition, some students (*n* = 15, 12%) found the feedback folio to be “onerous” or “not useful.”

### Faculty Focus Groups

A total of six faculty members participated in the focus group, representing the three sites. The discussion revealed two main themes: repetition as a means of improving feedback, and barriers to effective feedback exchange between student and teacher.

All participants agreed that the frequency of feedback increased due to the many reminders embedded in the curriculum about providing feedback (i.e., the faculty badges, posters, students needing to complete their folios, and email reminders). One participant said, “The badges triggered me to think about feedback whenever I saw them.” Another stated, “Making it part of what you do all the time makes for a safer environment to give and receive feedback.” There was also mention of repetition changing the type of feedback provided with one faculty commenting that the materials were a reminder to, “Make sure to say, ‘Good job doing X,’ instead of just saying, ‘Good job.’” Additionally, the daily recording of feedback in folios was a constant reminder for students to ask for feedback and faculty to provide it.

Another theme that emerged was barriers to effective feedback exchange between faculty and student. Participants discussed the iterative, long-term nature of feedback change saying, “It is like diet and exercise—it takes time, and you have to keep working at it.” Students’ unwillingness to receive difficult feedback and the competitive culture of students’ success depending on their performance were also seen as barriers to effective feedback exchange. There are competing forces when students say they want constructive feedback but then fear receiving it. One faculty member said, “Getting that constructive feedback feels so raw.” Another stated, “They are always thinking about the next step—performing at the next level,” when discussing students’ fear that feedback construed as negative will harm their ability to succeed in the future.

## Discussion

We successfully implemented a multipronged approach across three sites to improve the feedback exchange between student and teacher and change the learning environment culture within our OB/GYN departments. Our curriculum, targeting both faculty members and students, demonstrated that the Feedback Focused dual investment program resulted in daily feedback folio entries by students and increased frequency of feedback as reported by students on the end-of-clerkship evaluations. The majority of students perceived the feedback to be *adequate*. However, the percentage that reported faculty provide direction and constructive feedback *always* or *very often* remained roughly the same before and after implementing the program (69% vs. 70%, respectively). The majority of open-ended responses from the students were positive and highlighted the importance of teaching students to be proactive and self-directed in seeking feedback. Negative comments highlighted the need to establish meaningful relationships with faculty and residents to provide the foundation for feedback provision. Faculty reported the increase frequency of feedback was positive, but that students continued to fear constructive feedback due to the highly competitive nature of their training. The high percentage of students using the folios and sheer number of feedback entries demonstrated the practicality and successful implementation of the Feedback Focused program.

By educating learners about soliciting and recognizing feedback paired with faculty training on providing constructive and frequent feedback, we not only aimed to improve students’ perceptions of quantity and quality of feedback during the clerkship, but also aimed to create a feedback culture based on ongoing student-faculty interaction and trust in the feedback process. The development of our Feedback Focused curriculum program was grounded in the medical literature and implemented successfully at four large academic teaching hospitals on the OB/GYN clerkship. The teaching instruments and assessment methods were effective, easy to implement and utilize by both teachers and learners in our curriculum program, and proved to be sustainable over the year-long program period.

However, our feedback initiative and assessment of its effect had several limitations. The Feedback Focused program was conducted at four main teaching hospitals affiliated with a single medical school and in the specialty of OB/GYN, and thus the program specifics and outcomes may not be generalizable to other settings or specialties. The challenge of sustainability required constant vigilance and multiple reminders by the OB/GYN clerkship leadership at all teaching sites for both the teachers and learners to continue the program with each rotation. To implement this curriculum at a smaller institution with fewer resources alternative methods could be employed, including sending materials via email instead of printing, prerecording faculty and student development sessions, and setting up automatic, weekly email reminders for students and faculty. Other practical challenges included finding a champion for the program at each site, asking a more robust assessment question to students regarding why or why not the feedback was constructive, and identifying barriers to 100% student completion of feedback folios.

While our program was successful in improving the quantity of feedback received by students, the perception of the quality of feedback was not impacted. The evaluations highlighted the need for further faculty development on providing constructive and actionable feedback. In addition, short exposure to faculty and residents posed a barrier to creating meaningful relationships and thus effective feedback as perceived by the students. This is a culture change we hope to create through improved longitudinal relationships with faculty in ongoing efforts within our departments, and once adapted becomes easier to maintain over time.

Based on student suggestions, future iterations of the program will include a trial of the feedback folio in an online or app-enabled format. Future evaluations will include investigating the long-term effects of our program on student behavior by comparing AAMC GQ responses pre- and postimplementation. We will also continue to analyze data from the feedback folio entries, as ongoing feedback assessment has established importance.^22^ We believe that the Feedback Focused dual investment program developed and implemented within the OB/GYN clerkship will provide teachers and learners a lifelong skill in incorporating feedback into their professional lives within academic medicine.

## Appendices

Instructor Guide Faculty Session.docxVideo for Faculty.docxFaculty Badges.docxFolio Template.xlsxSlogan & Logo.docxFeedback Focused Posters.docxInstructor Guide Student Session.docxModule for Learners.pptxLearner Tips Card.docxEvaluation Form.docxFocus Group Questions.docx
All appendices are peer reviewed as integral parts of the Original Publication.
