# Supplementary material for: Feedback Focused: A Learner- and Teacher-Centered Curriculum to Improve the Feedback Exchange in the Obstetrics and Gynecology Clerkship
Source: MedEdPORTAL. 2021 Mar 25;17:11127. doi: 10.15766/mep_2374-8265.11127 (PMC8015633; doi:10.15766/mep_2374-8265.11127)
Supplement: Supplementary file 1 — Instructor Guide Faculty Session.docxVideo for Faculty.docxFaculty Badges.docxFolio Template.xlsxSlogan & Logo.docxFeedback Focused Posters.docxInstructor Guide Student Session.docxModule for Learners.pptxLearner Tips Card.docxEvaluation Form.docxFocus Group Questions.docx [file mep_2374-8265.11127-s001.zip › A. Instructor Guide Faculty Session.docx]

**Instructor’s Guide to the Faculty Learner Development Session**

Venue: Grand rounds, faculty meetings, departmental division meetings, resident didactics, educational retreats, or one-on-one meetings with individual faculty

Learners: All faculty members in OBGYN Department

Facilitators: Clerkship directors

Objectives:

Introduce the impetus and goals for the program and train/review how to provide constructive feedback:

1. Review student dissatisfaction with quantity and quality of feedback provided during training
2. Understand all components and goals of the intervention
3. Know how to provide constructive feedback

Session Content (approximately 20-25 minutes):

| Impetus for program | 3 minutes |
| --- | --- |
| Scope of the problem | 3 minutes |
| Goal of the program | 1 minutes |
| Description of the intervention | 3 minutes |
| Training on Constructive feedback | 8 minutes |
| Wrap up | 5 minutes |

1. Describe the **impetus** for faculty and student focused feedback intervention at your institution
   1. Students want more feedback
   2. Students do not feel general compliments are useful feedback
   3. Feedback leads to improved performance
   4. Positive cultural change
2. Present the **scope** of the problem
   1. Describe background of the importance of feedback including why it is important to train both the learner and the teacher for a successful intervention
   2. Present past or current data on student or faculty perceptions of feedback at your institution if available
      1. We used data from end of clerkship evaluations and institution based Association of the American Medical Colleges (AAMC) data
3. Share the **goal** of the intervention: to improve frequency and constructive nature of feedback during an OBGYN Clerkship through a bidirectional approach
4. Provide **description** of the program
   1. Explain the faculty badges and posters
   2. List all components of the student learner training
   3. Explain Feedback folios for learners
   4. Ongoing reminders about the Feedback Focused intervention – Say the F word often!
5. Provide training points on what **constructive feedback** is: Direct observation to improve a trainee’s performance
   1. Cover the following components:

- Label it feedback
- Determine an appropriate *location* or *setting* up front
- Focus on the behavior (limit to 2-3 observations)
- Make feedback goal oriented
- Be concrete and specific
- Praise in public, critique in private
- Be timely but wait until everyone is calm
- Be supportive
  1. Ask faculty to watch the APGO *Effective Preceptor Series: Providing Educational Feedback* video on their own time (Appendix B or we embed into this appendix)

Wrap up: time for questions

Materials: Slide containing data on student satisfaction with feedback (use data from your own institution or use national, publicly available data from AAMC GQ (https://www.aamc.org/data-reports/students-residents/report/graduation-questionnaire-gq) , APGO *Effective Preceptor Series: Providing Educational Feedback* video (Appendix B)
