# Supplementary material for: Feedback Focused: A Learner- and Teacher-Centered Curriculum to Improve the Feedback Exchange in the Obstetrics and Gynecology Clerkship
Source: MedEdPORTAL. 2021 Mar 25;17:11127. doi: 10.15766/mep_2374-8265.11127 (PMC8015633; doi:10.15766/mep_2374-8265.11127)
Supplement: Supplementary file 1 — Instructor Guide Faculty Session.docxVideo for Faculty.docxFaculty Badges.docxFolio Template.xlsxSlogan & Logo.docxFeedback Focused Posters.docxInstructor Guide Student Session.docxModule for Learners.pptxLearner Tips Card.docxEvaluation Form.docxFocus Group Questions.docx [file mep_2374-8265.11127-s001.zip › C. Faculty Badges.docx]

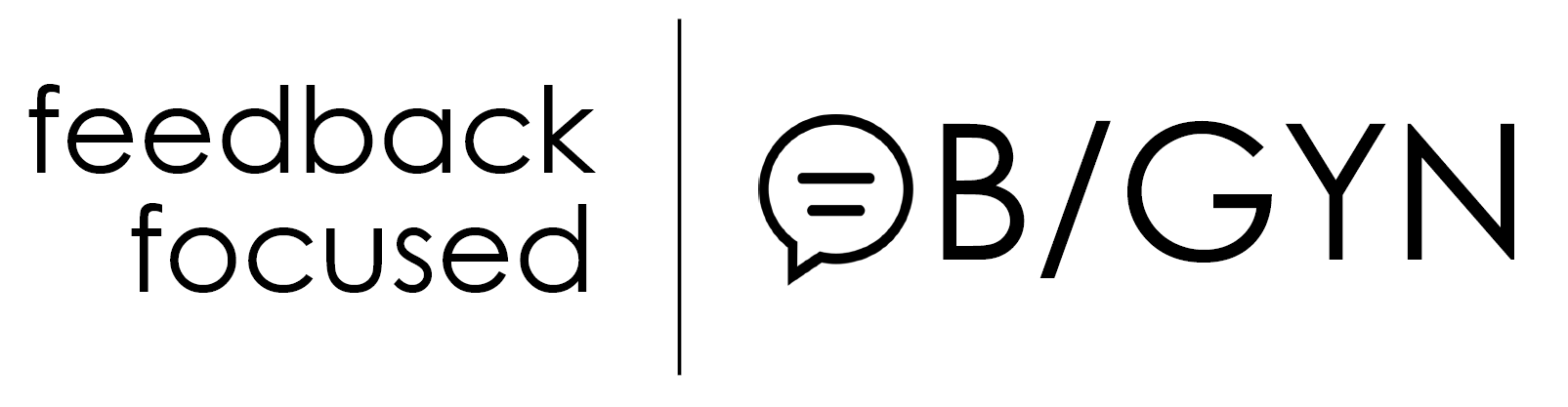


**Feedback 101:** **Feedback is our job**

- *Label* it **FEEDBACK!**
- Determine an appropriate *location* or *setting* up front
- Focus on the behavior (limit to 2-3 observations)
- Be concrete and specific
- Praise in public, critique in private
- Be timely but wait until everyone is calm
- Be supportive

*Teacher Cards (Back)*


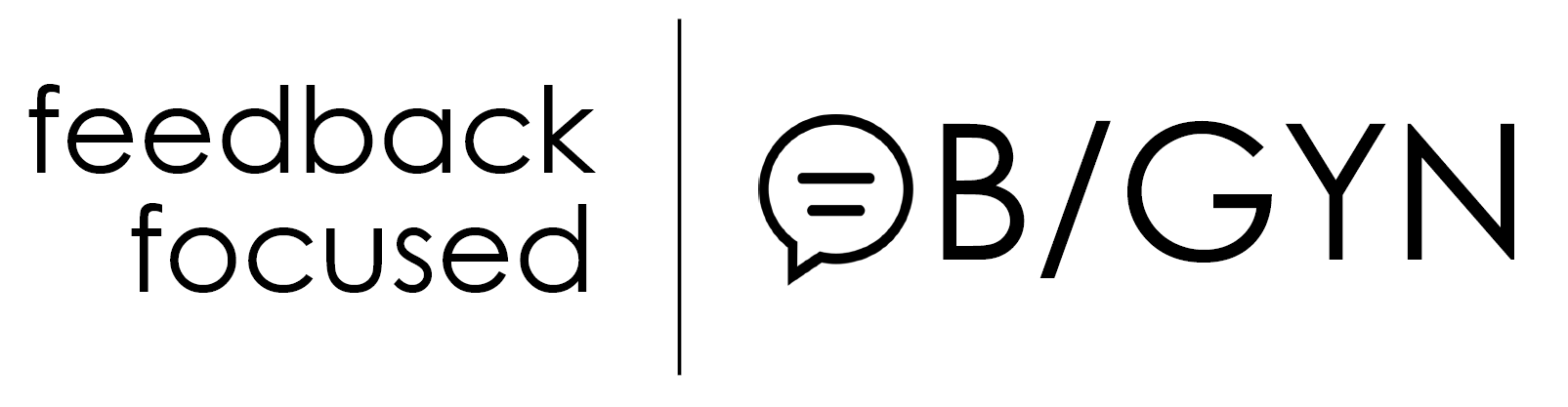


**Helpful Feedback Scripts:**

“It is my job to provide you constructive feedback”

“Let’s take a few minutes so I can give you some feedback.”

“I saw that you did ____. What else did you consider?”

“Some of this feedback may be disappointing, but I am here to help you address it.”

“So I am hearing you say ___. Can you say more about that?”
