# Supplementary material for: Feedback Focused: A Learner- and Teacher-Centered Curriculum to Improve the Feedback Exchange in the Obstetrics and Gynecology Clerkship
Source: MedEdPORTAL. 2021 Mar 25;17:11127. doi: 10.15766/mep_2374-8265.11127 (PMC8015633; doi:10.15766/mep_2374-8265.11127)
Supplement: Supplementary file 1 — Instructor Guide Faculty Session.docxVideo for Faculty.docxFaculty Badges.docxFolio Template.xlsxSlogan & Logo.docxFeedback Focused Posters.docxInstructor Guide Student Session.docxModule for Learners.pptxLearner Tips Card.docxEvaluation Form.docxFocus Group Questions.docx [file mep_2374-8265.11127-s001.zip › G. Instructor Guide Student Session.docx]

**Instructor’s Guide to the Student Learner Development Session**

Venue: Student orientation

Learners: Students entering OBGYN clerkship at start of each rotation

Facilitators: Clerkship directors

Objectives:

Introduce the impetus and goals for the program and teach how solicit, recognize, and utilize feedback:

1. Share motivation for initiative based on student feedback regarding faculty providing direction and constructive feedback
2. Describe the goals of the intervention and what is expected of the students
3. Teach techniques to effectively solicit feedback

Session Content (approximately 15-20 minutes):

| Motivation for program | 2 minutes |
| --- | --- |
| Goals of the program | 2 minutes |
| Description of the intervention | 2 minutes |
| Student expectations | 2 minutes |
| Training on soliciting, recognizing, utilizing feedback | 8 minutes |
| Wrap up | 2 minutes |

1. Describe the **motivation** for faculty and student focused feedback intervention at your institution
   1. Students want more feedback
      1. Present slide using data from your institution or from publicly available national data from AAMC GQ (https://www.aamc.org/data-reports/students-residents/report/graduation-questionnaire-gq)
   2. Students do not feel general compliments are useful feedback
   3. Feedback leads to improved performance
   4. Positive cultural change
2. Share the **goal** of the intervention: to improve frequency and constructive nature of feedback during an OBGYN Clerkship through a student and faculty centered approach
3. Provide **description** of the program
   1. Explain the feedback folios, student tip cards and posters
   2. Review the faculty learner component of the program
   3. Show logo to communicate the brand and dedication to being feedback focused
   4. Weekly reminders about recording feedback once per day
4. Explain **student expectation**:
   1. Record in the feedback folio at least once per day
   2. Think about your role in effective feedback relationship (solicit, recognize, and utilize)
   3. Review the feedback folios with clerkship director at mid- and end-of-clerkship evaluations
   4. Return the feedback folio at the end of the clerkship during your eld of clerkship evaluation
   5. Answer questions pertaining to the intervention on the end of clerkship evaluation survey
5. Show the Power point module *How to Effectively* ***Recognize, Solicit and Utilize Feedback*** *as a Medical Student*
   1. Utilize the notes on the notes page of each slide to guide your presentation and discussion

Wrap up: time for questions

Materials: Slide containing data on student satisfaction with feedback (use data from your own institution or use publicly available national data from AAMC GQ, Power point module *How to Effectively* ***Recognize, Solicit and Utilize Feedback*** *as a Medical Student*
