# Supplementary material for: Feedback Focused: A Learner- and Teacher-Centered Curriculum to Improve the Feedback Exchange in the Obstetrics and Gynecology Clerkship
Source: MedEdPORTAL. 2021 Mar 25;17:11127. doi: 10.15766/mep_2374-8265.11127 (PMC8015633; doi:10.15766/mep_2374-8265.11127)
Supplement: Supplementary file 1 — Instructor Guide Faculty Session.docxVideo for Faculty.docxFaculty Badges.docxFolio Template.xlsxSlogan & Logo.docxFeedback Focused Posters.docxInstructor Guide Student Session.docxModule for Learners.pptxLearner Tips Card.docxEvaluation Form.docxFocus Group Questions.docx [file mep_2374-8265.11127-s001.zip › H. Module for Learners.pptx]

## Slide 1
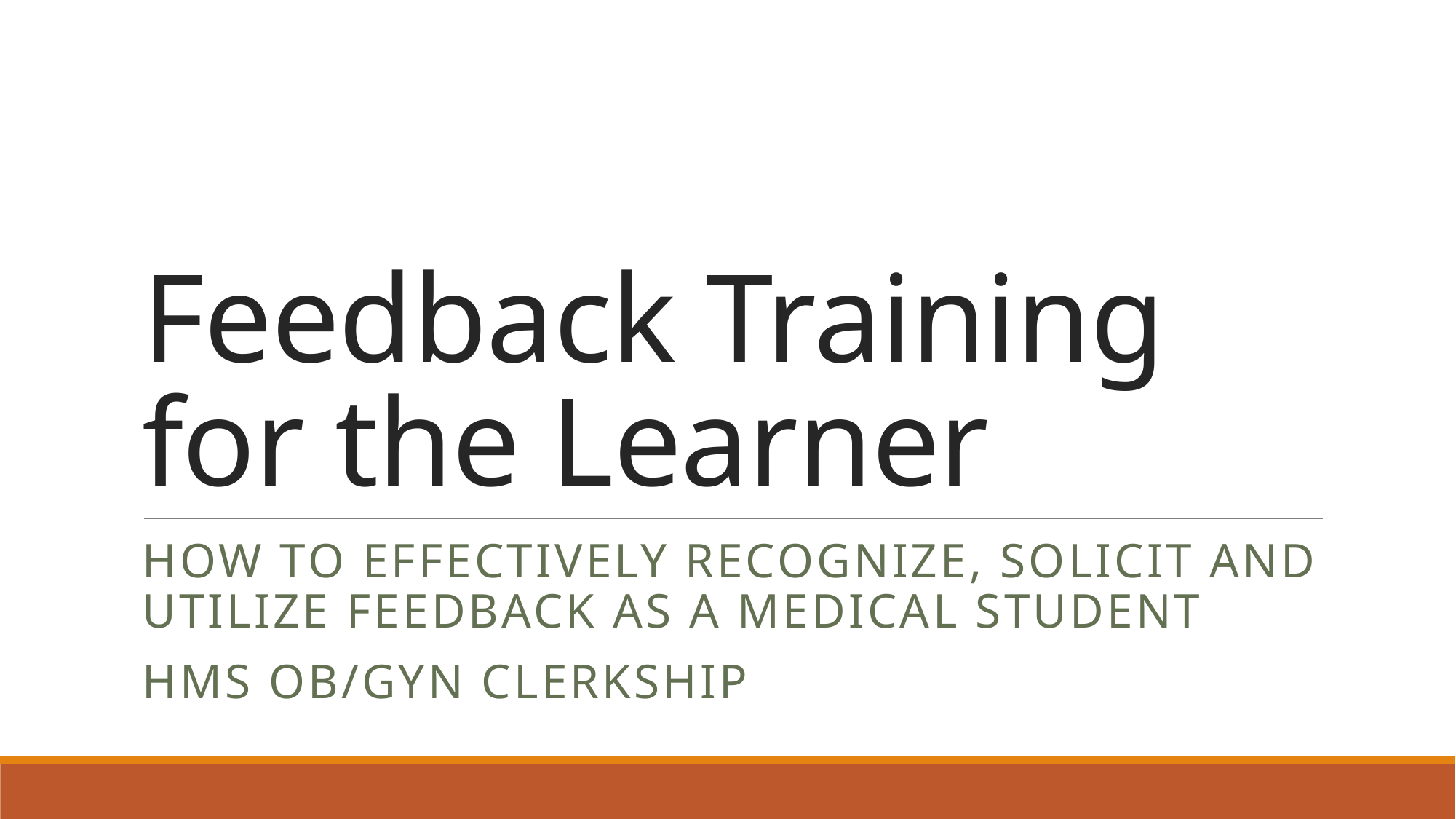

# Feedback Training for the Learner
How to Effectively Recognize, Solicit and Utilize Feedback as a Medical Student
HMS OB/gyn clerkship

## Slide 2
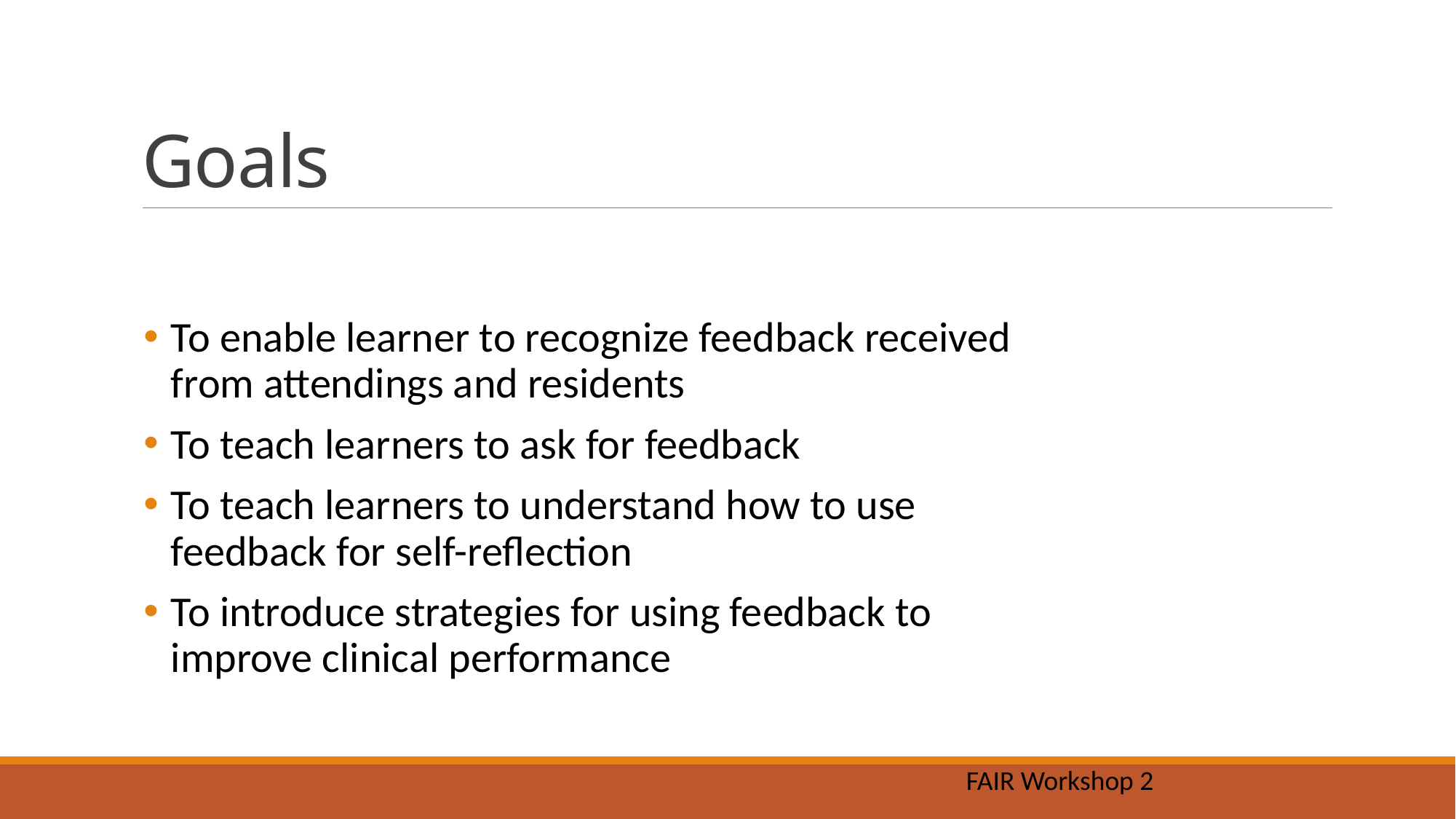

# Goals
To enable learner to recognize feedback received from attendings and residents
To teach learners to ask for feedback
To teach learners to understand how to use feedback for self-reflection
To introduce strategies for using feedback to improve clinical performance
FAIR Workshop 2

## Slide 3
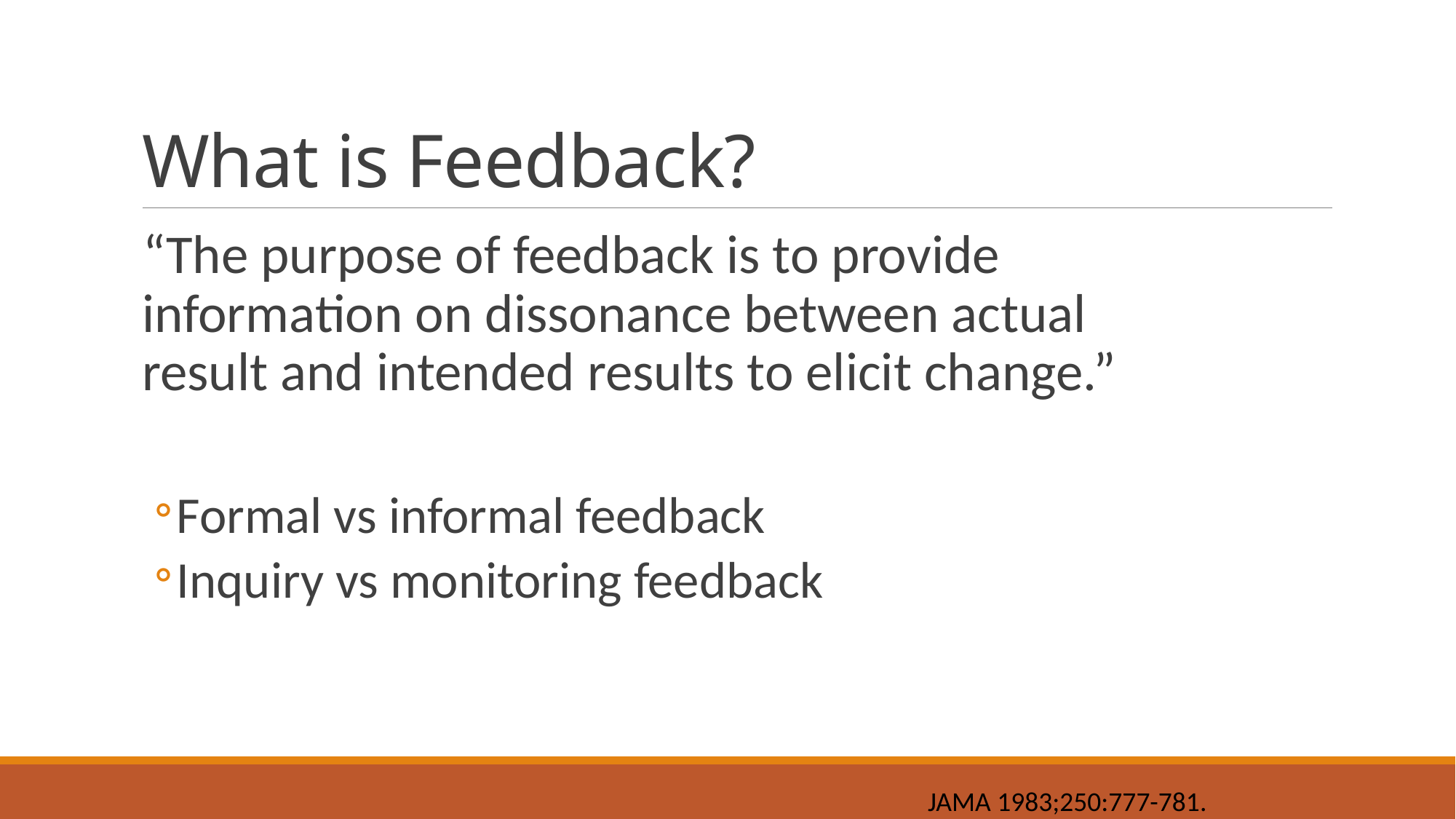

# What is Feedback?
“The purpose of feedback is to provide information on dissonance between actual result and intended results to elicit change.”
Formal vs informal feedback
Inquiry vs monitoring feedback
JAMA 1983;250:777-781.

## Slide 4
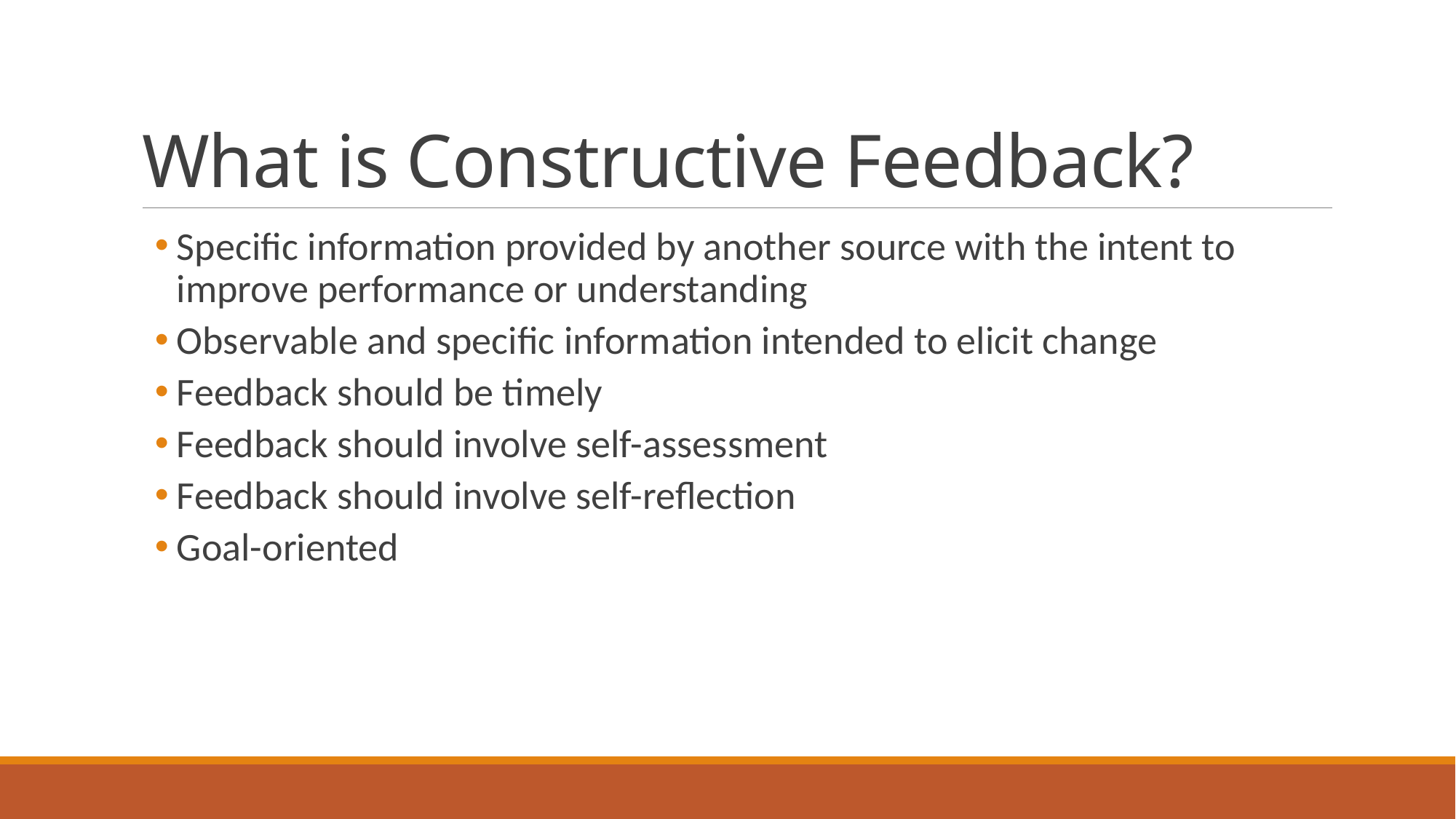

# What is Constructive Feedback?
Specific information provided by another source with the intent to improve performance or understanding
Observable and specific information intended to elicit change
Feedback should be timely
Feedback should involve self-assessment
Feedback should involve self-reflection
Goal-oriented

## Slide 5
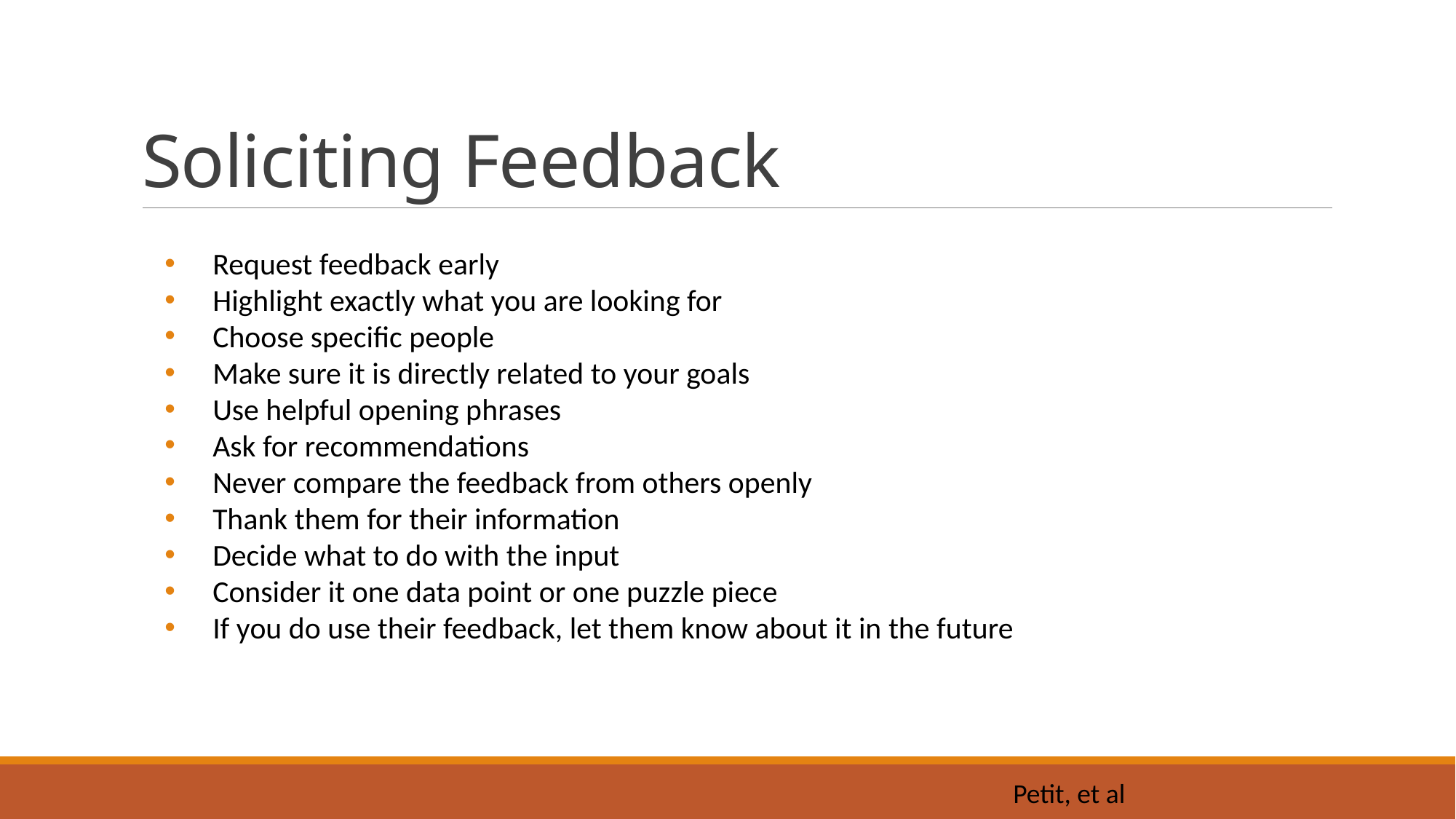

# Soliciting Feedback
 Request feedback early
 Highlight exactly what you are looking for
 Choose specific people
 Make sure it is directly related to your goals
 Use helpful opening phrases
 Ask for recommendations
 Never compare the feedback from others openly
 Thank them for their information
 Decide what to do with the input
 Consider it one data point or one puzzle piece
 If you do use their feedback, let them know about it in the future
Petit, et al

## Slide 6
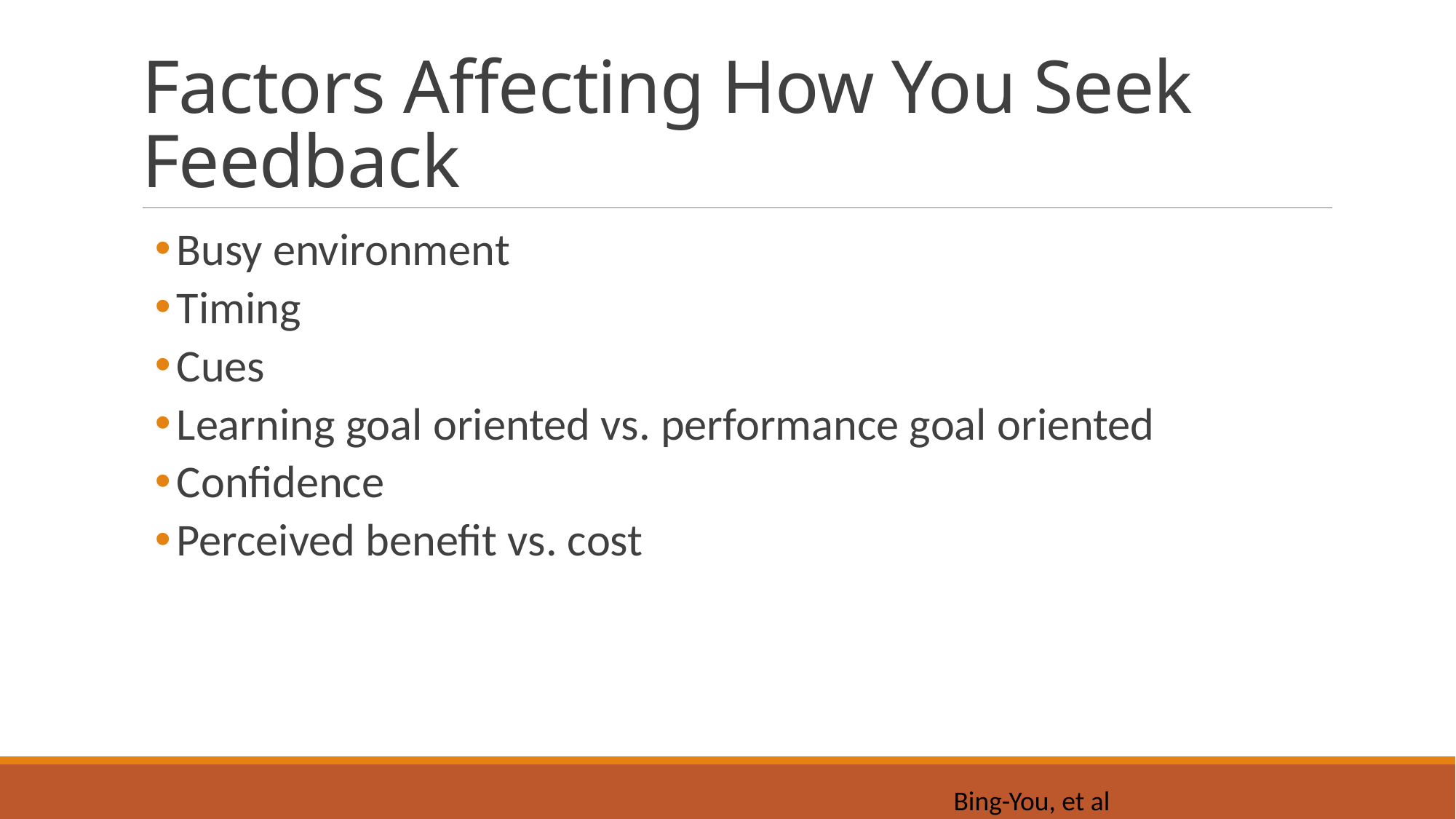

# Factors Affecting How You Seek Feedback
Busy environment
Timing
Cues
Learning goal oriented vs. performance goal oriented
Confidence
Perceived benefit vs. cost
Bing-You, et al

## Slide 7
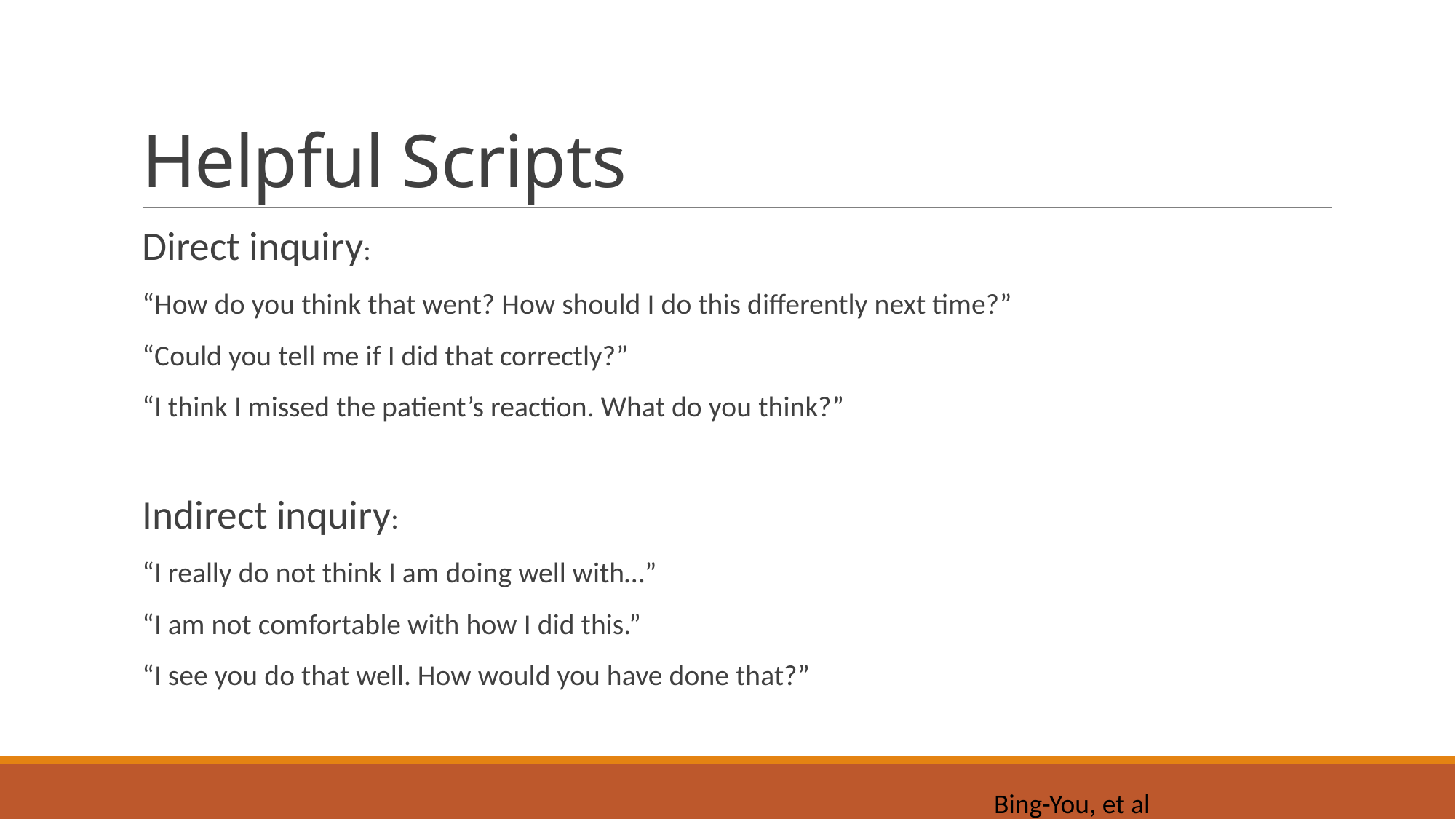

# Helpful Scripts
Direct inquiry:
“How do you think that went? How should I do this differently next time?”
“Could you tell me if I did that correctly?”
“I think I missed the patient’s reaction. What do you think?”
Indirect inquiry:
“I really do not think I am doing well with…”
“I am not comfortable with how I did this.”
“I see you do that well. How would you have done that?”
Bing-You, et al

## Slide 8
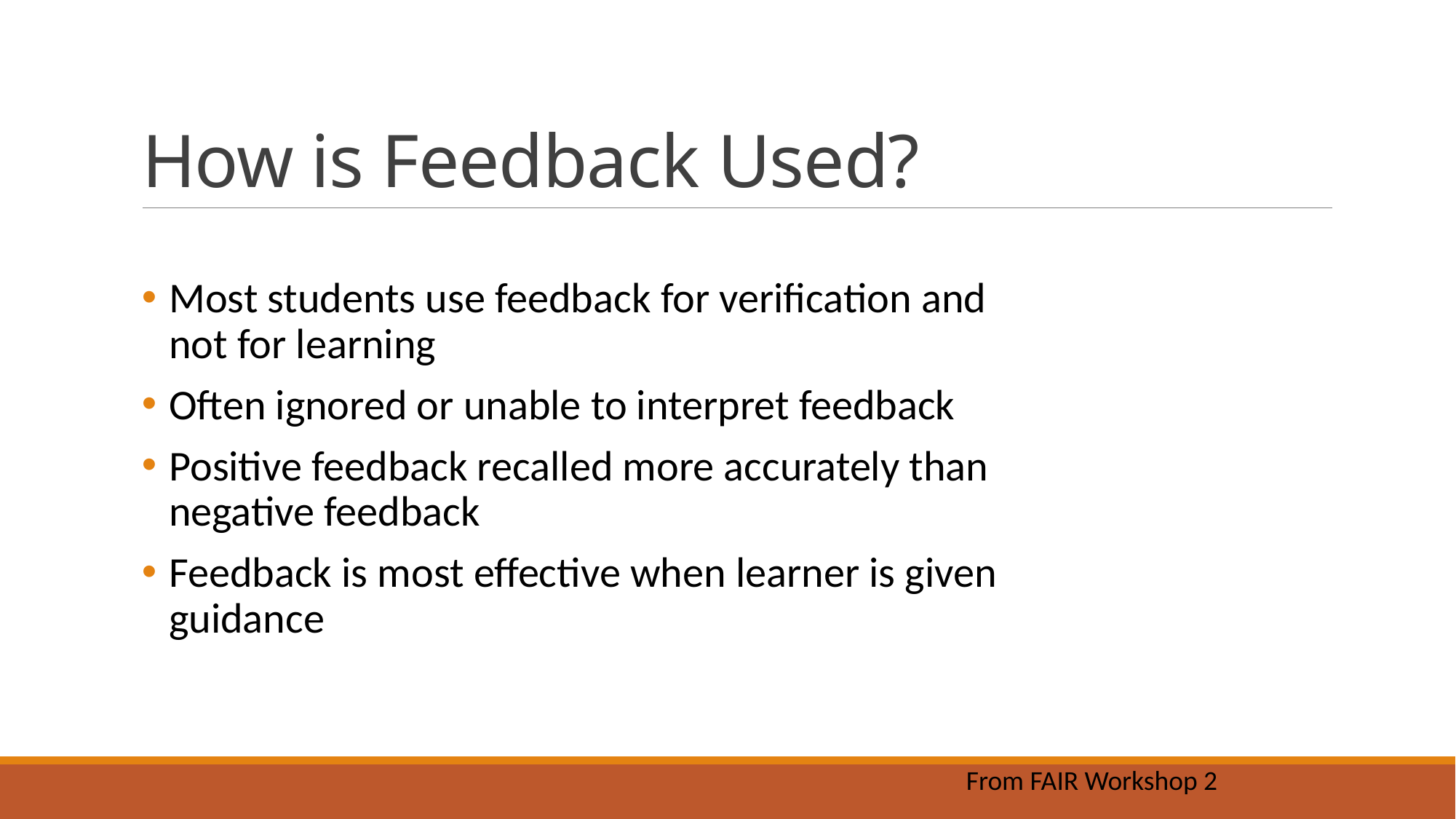

# How is Feedback Used?
Most students use feedback for verification and not for learning
Often ignored or unable to interpret feedback
Positive feedback recalled more accurately than negative feedback
Feedback is most effective when learner is given guidance
From FAIR Workshop 2

## Slide 9
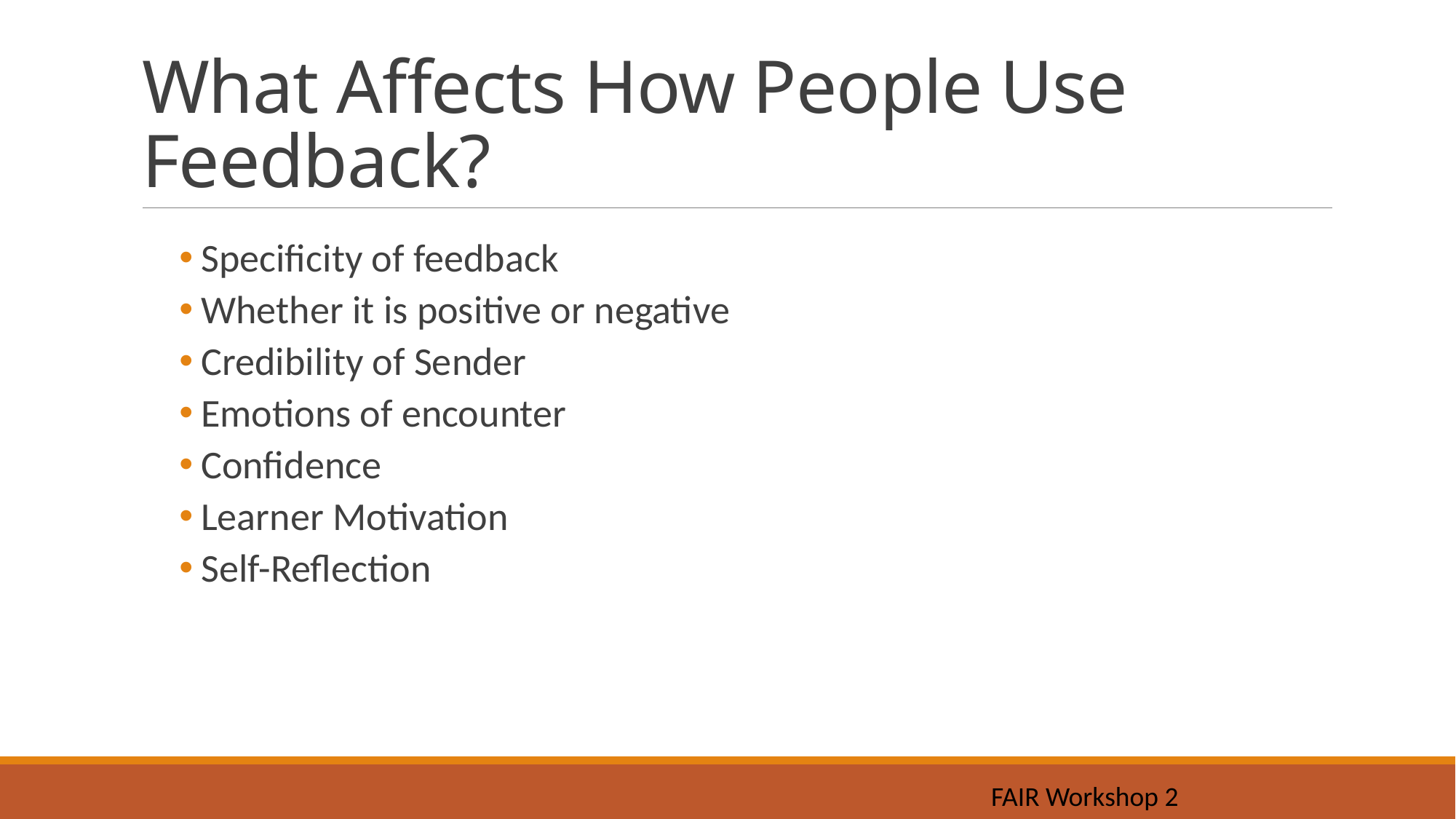

# What Affects How People Use Feedback?
Specificity of feedback
Whether it is positive or negative
Credibility of Sender
Emotions of encounter
Confidence
Learner Motivation
Self-Reflection
FAIR Workshop 2

## Slide 10
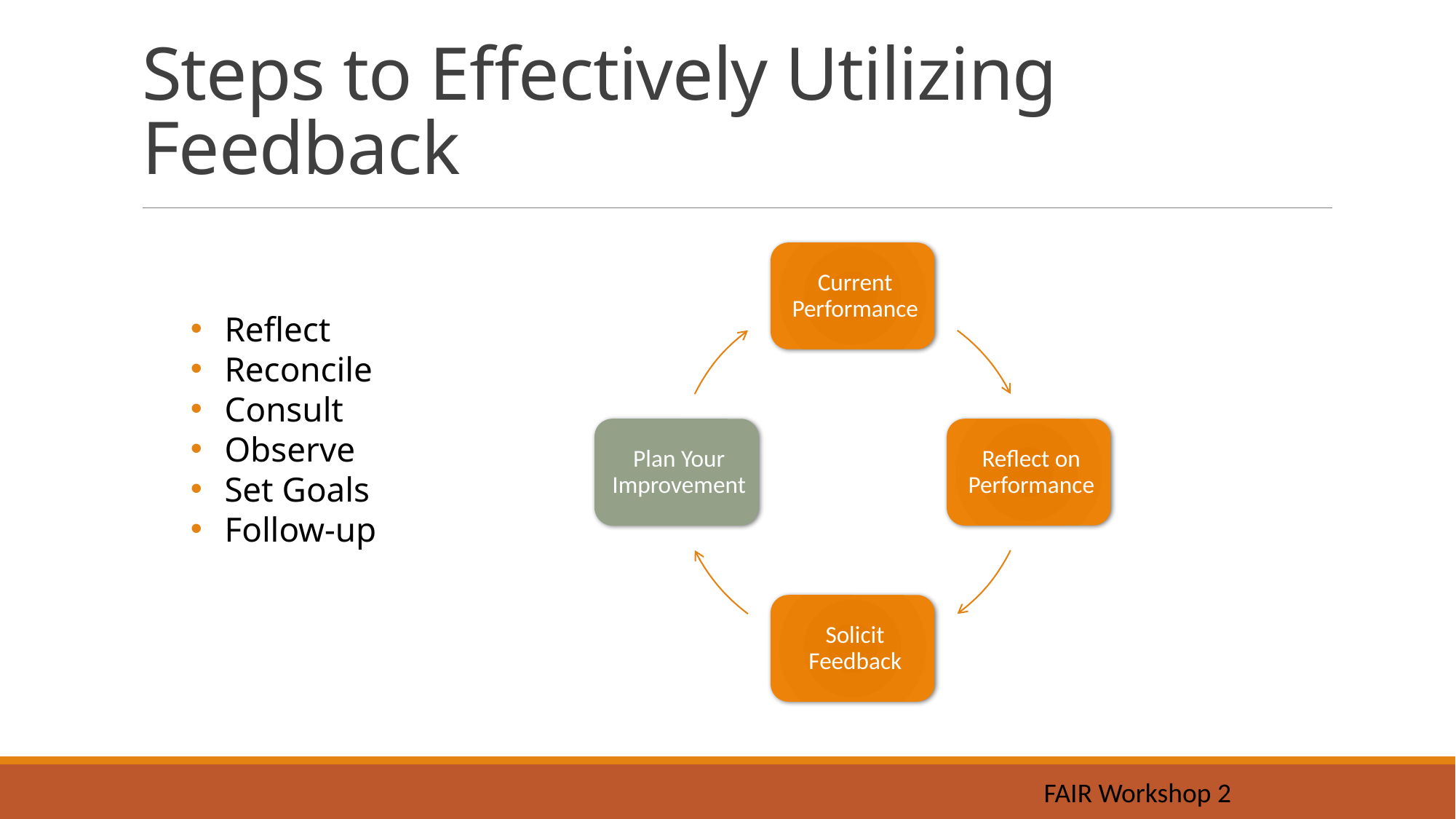

# Steps to Effectively Utilizing Feedback
Reflect
Reconcile
Consult
Observe
Set Goals
Follow-up
FAIR Workshop 2

## Slide 11
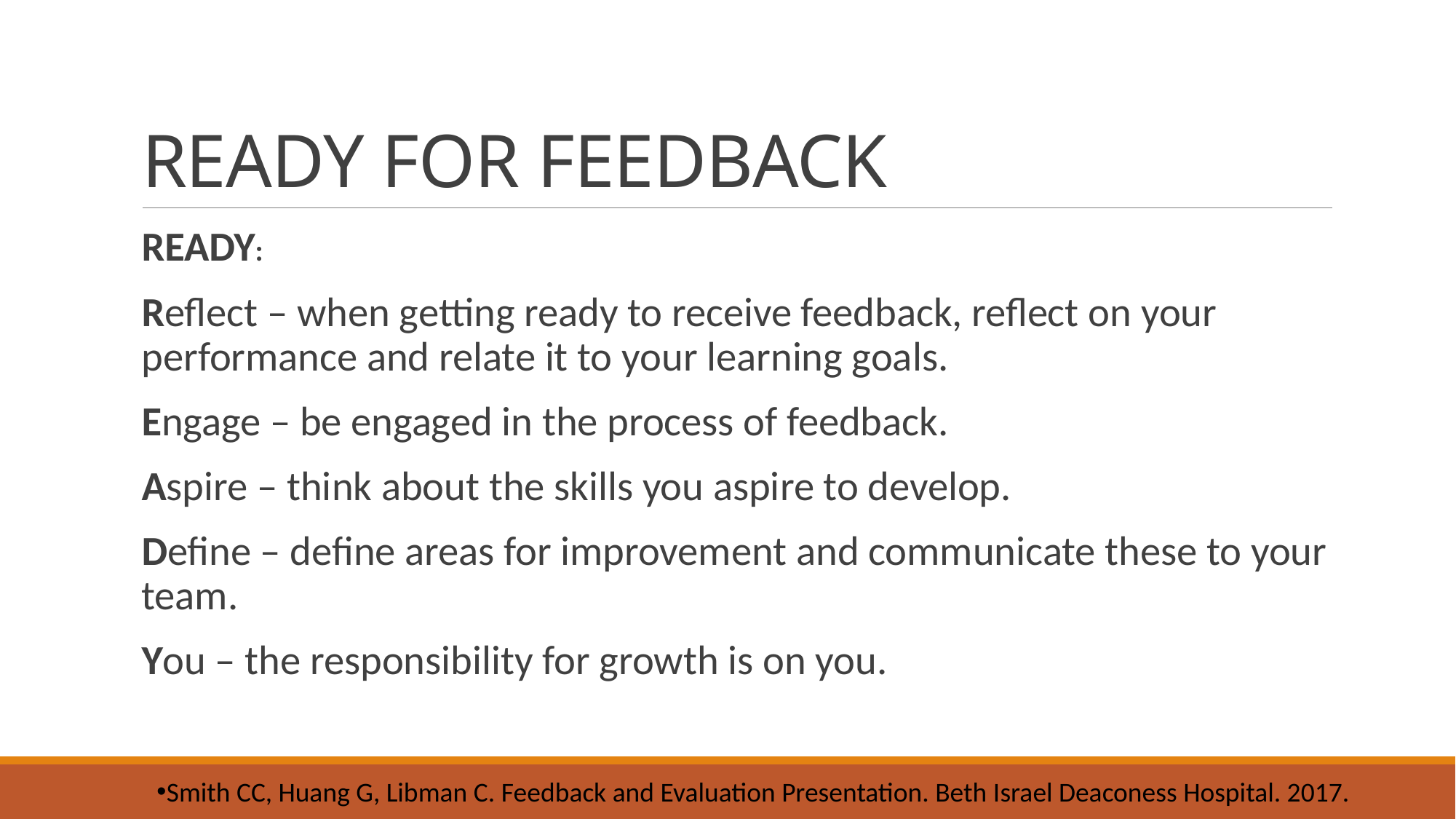

# READY FOR FEEDBACK
READY:
Reflect – when getting ready to receive feedback, reflect on your performance and relate it to your learning goals.
Engage – be engaged in the process of feedback.
Aspire – think about the skills you aspire to develop.
Define – define areas for improvement and communicate these to your team.
You – the responsibility for growth is on you.
Smith CC, Huang G, Libman C. Feedback and Evaluation Presentation. Beth Israel Deaconess Hospital. 2017.

## Slide 12
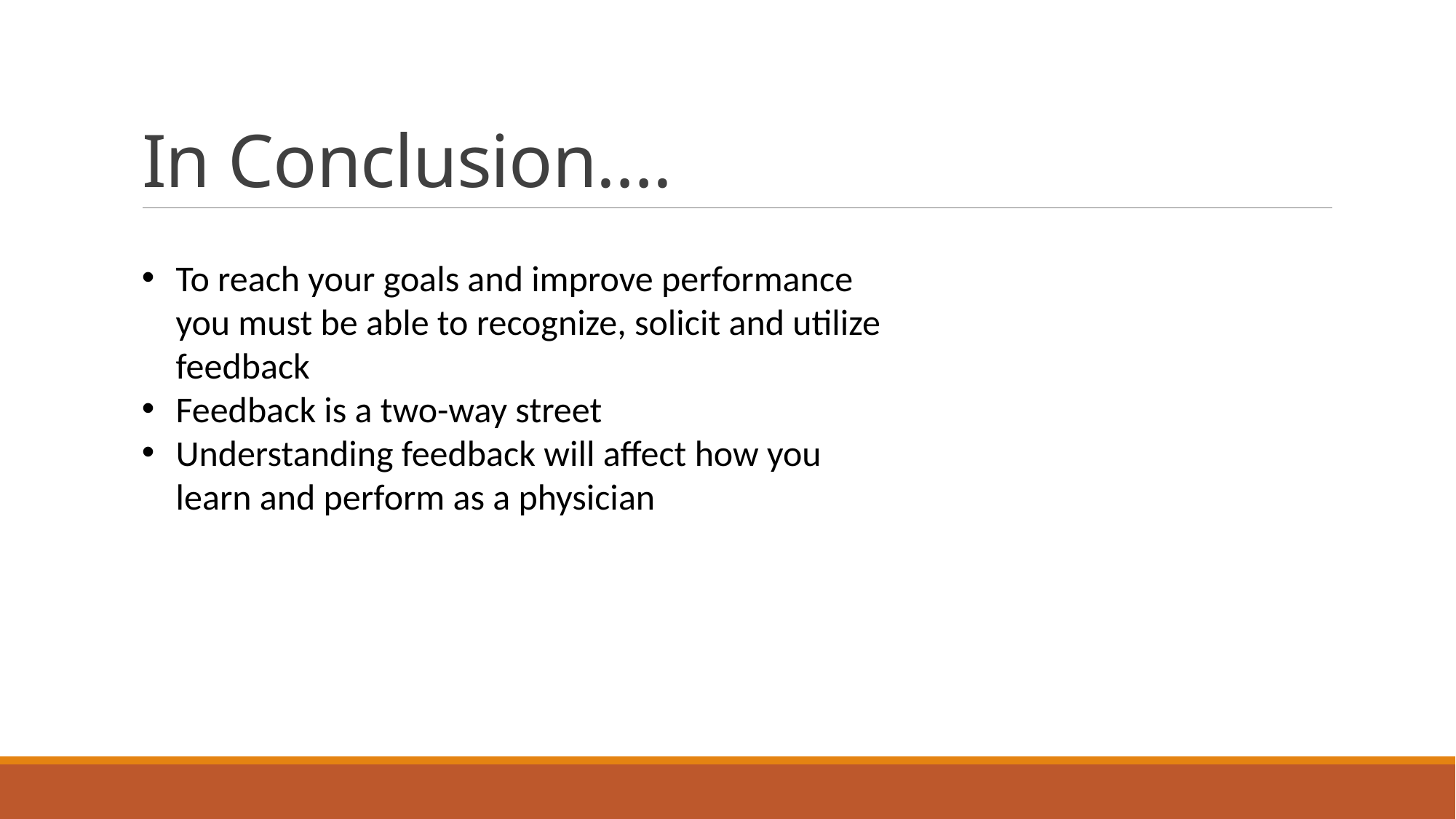

# In Conclusion….
To reach your goals and improve performance you must be able to recognize, solicit and utilize feedback
Feedback is a two-way street
Understanding feedback will affect how you learn and perform as a physician

## Slide 13
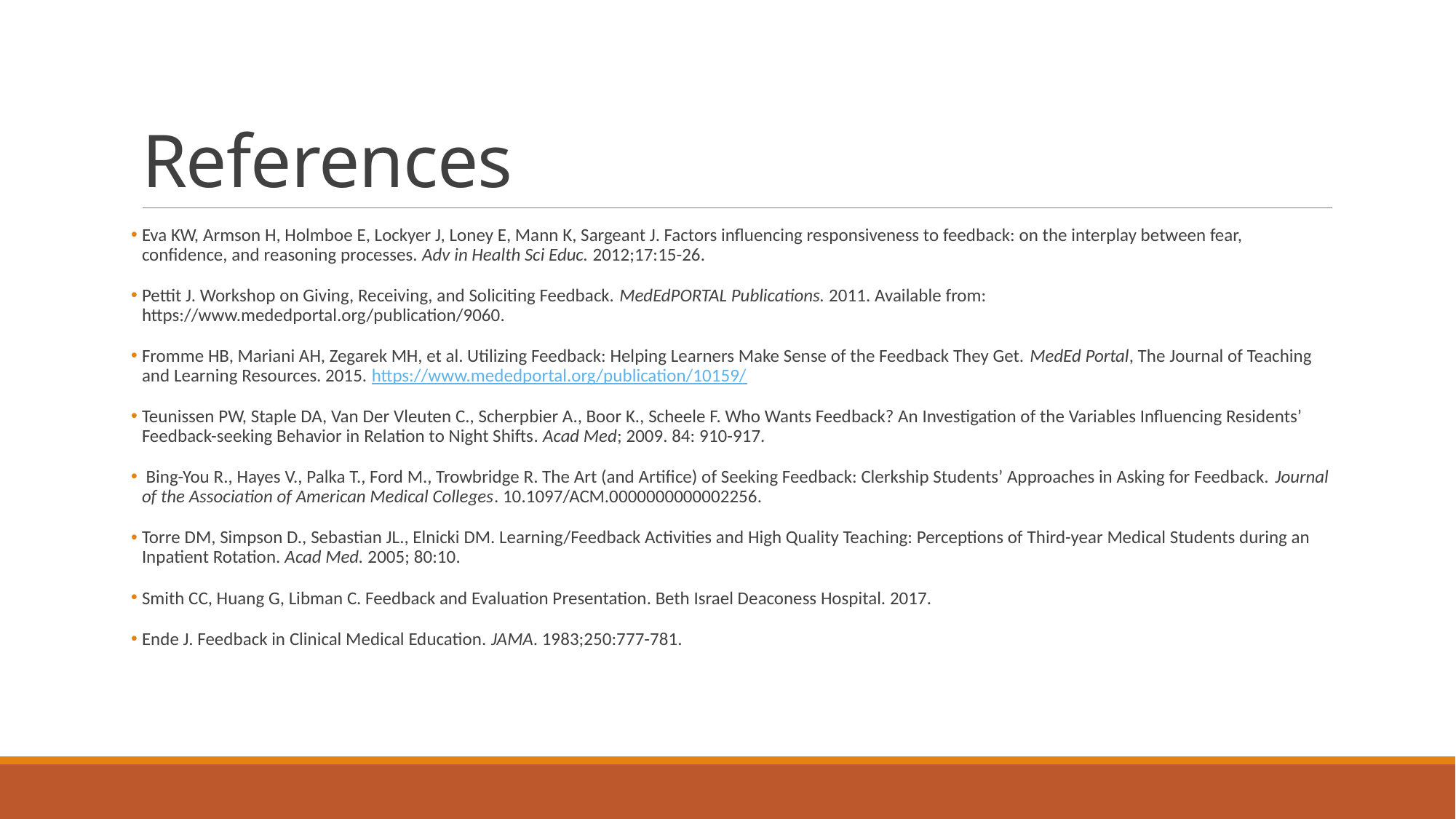

# References
Eva KW, Armson H, Holmboe E, Lockyer J, Loney E, Mann K, Sargeant J. Factors influencing responsiveness to feedback: on the interplay between fear, confidence, and reasoning processes. Adv in Health Sci Educ. 2012;17:15-26.
Pettit J. Workshop on Giving, Receiving, and Soliciting Feedback. MedEdPORTAL Publications. 2011. Available from: https://www.mededportal.org/publication/9060.
Fromme HB, Mariani AH, Zegarek MH, et al. Utilizing Feedback: Helping Learners Make Sense of the Feedback They Get. MedEd Portal, The Journal of Teaching and Learning Resources. 2015. https://www.mededportal.org/publication/10159/
Teunissen PW, Staple DA, Van Der Vleuten C., Scherpbier A., Boor K., Scheele F. Who Wants Feedback? An Investigation of the Variables Influencing Residents’ Feedback-seeking Behavior in Relation to Night Shifts. Acad Med; 2009. 84: 910-917.
 Bing-You R., Hayes V., Palka T., Ford M., Trowbridge R. The Art (and Artifice) of Seeking Feedback: Clerkship Students’ Approaches in Asking for Feedback. Journal of the Association of American Medical Colleges. 10.1097/ACM.0000000000002256.
Torre DM, Simpson D., Sebastian JL., Elnicki DM. Learning/Feedback Activities and High Quality Teaching: Perceptions of Third-year Medical Students during an Inpatient Rotation. Acad Med. 2005; 80:10.
Smith CC, Huang G, Libman C. Feedback and Evaluation Presentation. Beth Israel Deaconess Hospital. 2017.
Ende J. Feedback in Clinical Medical Education. JAMA. 1983;250:777-781.
