# Supplementary material for: Feedback Focused: A Learner- and Teacher-Centered Curriculum to Improve the Feedback Exchange in the Obstetrics and Gynecology Clerkship
Source: MedEdPORTAL. 2021 Mar 25;17:11127. doi: 10.15766/mep_2374-8265.11127 (PMC8015633; doi:10.15766/mep_2374-8265.11127)
Supplement: Supplementary file 1 — Instructor Guide Faculty Session.docxVideo for Faculty.docxFaculty Badges.docxFolio Template.xlsxSlogan & Logo.docxFeedback Focused Posters.docxInstructor Guide Student Session.docxModule for Learners.pptxLearner Tips Card.docxEvaluation Form.docxFocus Group Questions.docx [file mep_2374-8265.11127-s001.zip › J. Evaluation Form.docx]

**Preview Evaluation**

**2019-2020 : PCE BWH : 103-OB600M.23 - Core Clerkship Obstetrics/Gynecology**

| **Course Evaluation** | | | | |
| --- | --- | --- | --- | --- |
| **HMS OBGYN Clerkship Evaluation - UPDATED** | | | | |
| **Course Information** | | | | |
| **Date** | **Course** | **Location** | **Weeks** | **Credits** |
|  | XXX-YYY: Department Course | Location |  |  |
| **Evaluation Period:** | | | | |
| This is a standard set of questions used by students to evaluate core OBGYN clerkships at Harvard Medical School (HMS). Your responses to these questions are essential to monitor the quality of  the educational experience at the Medical School. The identity of all respondents is confidential. | | | | |

**Please rate how often the following professional behaviors/attitudes are demonstrated by Attending Physicians involved in your clerkship.**

Providing direction and constructive feedback.

- Never
- Almost never
- Sometimes
- Fairly often
- Very often
- Always

How many times per month did you receive feedback?

- 0
- 1
- 2
- 3
- 4+

Did you have an exit interview to summarize your performance?

- Yes
- No

Do you consider the frequency of feedback you received during your clerkship to be:

- Not enough
- Adequate amount
- Too much

We define constructive feedback as actionable: providing specifics based on an observation(s) with the intent to improve performance. Constructive feedback was provided to you on this clerkship:

- Always
- Often
- Rarely
- Never

What do you think about the overall program on this clerkship?
