# Supplementary material for: Feedback Focused: A Learner- and Teacher-Centered Curriculum to Improve the Feedback Exchange in the Obstetrics and Gynecology Clerkship
Source: MedEdPORTAL. 2021 Mar 25;17:11127. doi: 10.15766/mep_2374-8265.11127 (PMC8015633; doi:10.15766/mep_2374-8265.11127)
Supplement: Supplementary file 1 — Instructor Guide Faculty Session.docxVideo for Faculty.docxFaculty Badges.docxFolio Template.xlsxSlogan & Logo.docxFeedback Focused Posters.docxInstructor Guide Student Session.docxModule for Learners.pptxLearner Tips Card.docxEvaluation Form.docxFocus Group Questions.docx [file mep_2374-8265.11127-s001.zip › K. Focus Group Questions.docx]

**Feedback Focused: Faculty Feedback Questions for Focus Group**

1. Did you watch the APGO training video entitled: APGO Effective Preceptor Series: Providing Educational Feedback?
2. If yes, did the APGO training video provide any new information regarding providing constructive feedback?
3. Did awareness of providing quality feedback, through the program overview, badges, posters, and frequent reminders, affect how often you provided feedback? Why or why not?
4. Did awareness of providing quality feedback, through the program overview, badges, posters, and frequent reminders, affect the type of feedback you provided? Why or why not?
5. Do you think the feedback focused program improved the culture of providing frequent and constructive feedback in the ONGYN Clerkship?
6. Do you think the Feedback Focused Program improved the culture of students asking for feedback in the OBGYN Clerkship?
7. Do you think students more often asked for feedback compared to years prior to our program?
8. Do you think students were receptive to true, constructive feedback?
9. Can you give me an example of a time you utilized the teaching of feedback from the faculty development during grand rounds, faculty meetings, departmental division meetings, resident didactics, educational retreats, and one-on-one meetings in providing feedback to a student?
10. While students reported an increase if feedback frequency after the implementation of our program, students did not report “faculty providing direction and constructive feedback” more often. Please provide thoughts on why this might be.
11. Please provide any other thoughts about the Feedback Focused program.
