# Supplementary figures and images for: Feedback Focused: A Learner- and Teacher-Centered Curriculum to Improve the Feedback Exchange in the Obstetrics and Gynecology Clerkship
Source: MedEdPORTAL. 2021 Mar 25;17:11127. doi: 10.15766/mep_2374-8265.11127 (PMC8015633; doi:10.15766/mep_2374-8265.11127)

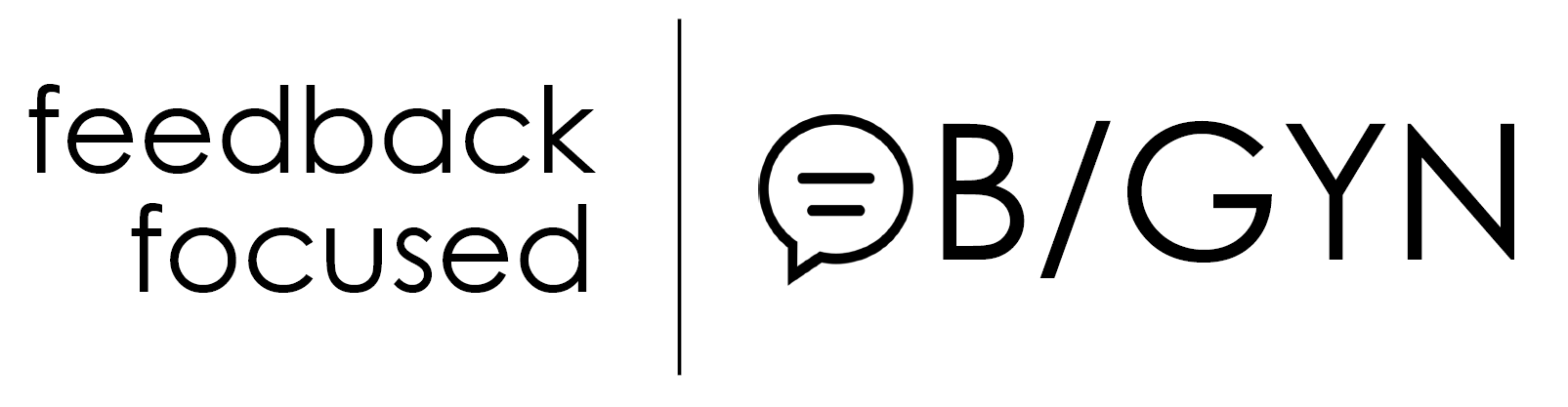


“Author owned”

Supplement: Supplementary file 1 — Instructor Guide Faculty Session.docxVideo for Faculty.docxFaculty Badges.docxFolio Template.xlsxSlogan & Logo.docxFeedback Focused Posters.docxInstructor Guide Student Session.docxModule for Learners.pptxLearner Tips Card.docxEvaluation Form.docxFocus Group Questions.docx [file mep_2374-8265.11127-s001.zip › E. Slogan & Logo.docx]
